# Treatment and Response Factors in Muscle Activation during Spinal Manipulation

**DOI:** 10.3390/jcm12196377

**Published:** 2023-10-06

**Authors:** Stuart J. Currie, Casey A. Myers, Brian A. Enebo, Bradley S. Davidson

**Affiliations:** 1 Independent Researcher, Sheridan, CO 80110, USA; 2 Department of Mechanical and Materials Engineering, University of Denver, 2155 East Wesley Ave, Denver, CO 80208, USA; casey.myers@du.edu (C.A.M.);; 3 Independent Researcher, Golden, CO 80403, USA

**Keywords:** spinal manipulation, reflex, paraspinal muscles, electromyography

## Abstract

The forces applied during a spinal manipulation produce a neuromuscular response in the paraspinal muscles. A systematic evaluation of the factors involved in producing this muscle activity provides a clinical insight. The purpose of this study is to quantify the effect of *treatment factors* (manipulation sequence and manipulation site) and *response factors* (muscle layer, muscle location, and muscle side) on the neuromuscular response to spinal manipulation. The surface and indwelling electromyographies of 8 muscle sites were recorded during lumbar side-lying manipulations in 20 asymptomatic participants. The effects of the factors on the number of muscle responses and the muscle activity onset delays were compared using mixed-model linear regressions, effect sizes, and equivalence testing. The *treatment factors* did not reveal statistical differences between the manipulation sequences (first or second) or manipulation sites (L3 or SI) in the number of muscle responses (*p* = 0.11, *p* = 0.28, respectively), or in muscle activity onset delays (*p* = 0.35 *p* = 0.35, respectively). There were significantly shorter muscle activity onset delays in the multifidi compared to the superficial muscles (*p* = 0.02). A small effect size of side (*d* = 0.44) was observed with significantly greater number of responses (*p* = 0.02) and shorter muscle activity onset delays (*p* < 0.001) in the muscles on the left side compared to the right. The location, layer, and side of the neuromuscular responses revealed trends of decreasing muscle response rates and increasing muscle activity onset delays as the distance from the manipulation site increased. These results build on the body of work suggesting that the specificity of manipulation site may not play a role in the neuromuscular response to spinal manipulation—at least within the lumbar spine. In addition, these results demonstrate that multiple manipulations performed in similar areas (L3 and S1) do not change the response significantly, as well as contribute to the clinical understanding that the muscle response rate is higher and with a shorter delay, the closer it is to the manipulation.

## 1. Introduction

Low back pain is the second most common cause for visits to a primary care physician [1], and accounts for billions of dollars in annual costs through medical expenses, missed work, reduced job performance [2], and is increasing significantly in prevalence [3]. Spinal manipulation is a cost-effective treatment when used alone or in combination with other techniques [4], and clinical practice guidelines recommend it as an accepted treatment for spine pain [5,6]. The forces produced during spinal manipulation (SM) have been shown to induce individual vertebral motion, increase facet joint gapping, and produce changes in intradiscal pressure, pain thresholds, and paraspinal muscle activity [7]. Changes in muscle activity, as measured by electromyography (EMG), are a measure of the neuromuscular response to SM, and may contribute to the mechanism of pain reduction [8,9]. However, measures of neuromuscular responses have produced inconsistent results across the research [10], which have shown both reductions [11] and excitatory effects [12] in resting EMG activity. Translating the basic scientific research on SM into a clinical setting can be challenging because factors that affect the experimental design and interpretation have not been systematically quantified. In the neuromuscular response to SM, two primary factor groups should be considered: (1) *treatment* and (2) *response*. *Treatment factors* are variables that are chosen by the practitioner, such as the manipulation sequence and manipulation site. *Response factors* are elements of the patient’s neuromuscular response, such as the muscle layer, muscle location, and side of the muscular response. Understanding the effects of these factors, which are rarely reported in SM literature, may help explain the inconsistent results observed across studies [10].

It is currently unclear how the neuromuscular response changes with multiple manipulations performed in a sequence. Study designs replicate how SM is delivered in a clinical setting by performing SM at multiple sites [13]. To maximize the time in the laboratory, considerations of equipment set up, time needed to data acquisition time, and participant comfort, often lead to study designs where multiple manipulations are performed. Sometimes as many as 16 treatments are performed at the same site [14]. In other cases, manipulations are repeated until a desirable result is achieved [13]. 

Understanding how the site of the applied manipulation force affects the neuromuscular response in healthy people helps inform both clinical delivery and research designs by establishing baseline values for future work in participants experiencing pain. The manipulation site may affect the muscles that are involved in neuromuscular responses [13]. In the clinic, a wide variety of methods are used to determine where to administer the SM [15], and a manipulation that is applied to a painful spinal segment results in muscle activity reductions that do not occur when applied to a non-painful spinal segment [16]. This finding of different responses at painful sites has led to an emphasis on measuring the response to SM in clinically relevant painful areas [10]. This approach has the advantage that the manipulation is applied to a clinically relevant area, but leaves the choice of location to the practitioner, which varies from participant to participant within a given study, and presents difficulties in directly comparing the results across participants where manipulation is performed at different sites. 

The interpretation of the neuromuscular response to manipulation is influenced by *response factors*, including the layer, location, and side of the responding muscle. The prevailing view of low back muscles is that the superficial layer of erector spinae muscles are broad movers of the trunk, while the deep layer of multifidi are responsible for segmental control [17]. In patients with LBP, reductions in multifidus activity were observed, which were not observed in the erector spinae [18]. These muscles performed different functions and behaved differently in the presence of LBP, underscoring the importance of delineating the effects of SM on these different muscle layers. The neuromuscular response to SM appears to depend on the distance of the muscle from the manipulation site [19,20]. Neuromuscular responses to SM have been found to exist in muscle locations anatomically distant from the manipulation site, but not uniformly across participants [13]. In feline specimens, the rate of muscle spindle firing increases as the manipulation is applied closer to the measurement location [21]. Spinal manipulation is often applied asymmetrically in the lumbar spine and sacroiliac (SI) joint, which results in different responses on the left and right sides of the body [13]. In addition, these side-to-side differences may depend on the manipulation side. SM applied to the left side has been shown to result in fewer responses than an equivalent treatment on the right side of the body, which remains unexplained [13].

Despite the recognized importance of the multifidus in low back biomechanics and pathology [17], activity *during* a spinal manipulation is not regularly recorded. Indwelling EMG is the method of choice for quantifying the activity of the multifidus [22]; however, it is not as easily recorded as more superficial paraspinal muscles. Spinal manipulation may alter neuromuscular activity in the paraspinal muscles [8], with pioneering work being performed in the erector spinae [13,23]. Increased erector spinae activity during a maximal voluntary contraction following SM compared to pre-SM activity was demonstrated in a clinical trial [24]; however, the effect of SM on the activity of the multifidus was limited to case reports. Increases in multifidus activity [25] and thickness [26,27] were demonstrated following SM, which implied a link to multifidus function. These case reports and studies of multifidus thickness provide the foundation for a comprehensive evaluation of the effect of SM on multifidus activity. 

The goal of the present study is to quantify the effects of treatment factors (manipulation sequence and manipulation site) and response factors (muscle layer, muscle location, and muscle side) on two neuromuscular variables that characterize the quantity and timing of the response. Eight muscles in two layers of the low back are recorded during a spinal manipulation. Quantifying the differences in these responses will lead to improved study design, better clinical delivery, and contribute to a better understanding of the mechanisms of this treatment.

Low back pain is the second most common cause for visits to a primary care physician [1], and accounts for billions of dollars in annual costs through medical expenses, missed work, and reduced job performance [2]. Spinal manipulation is a cost-effective treatment when used alone or in combination with other techniques [4], and is an accepted treatment for low back pain [5]. The forces produced during spinal manipulation (SM) have been shown to induce individual vertebral motion, increase facet joint gapping, and produce changes in intradiscal pressure, pain thresholds, and paraspinal muscle activity [7].

## 2. Materials and Methods

### 2.1. Participant Information

Twenty participants (Table 1), each with no history of low back pain during the previous four years, visited the laboratory for a single test session that lasted three to four hours, during which muscle activity was monitored in the low back during SM. Orthopedic and neurologic examinations were performed to screen the participants for contraindications to SM, including radicular pain below the knee, sensation, or weakness in the lower extremity, or a pain level that exceeded seven out of ten on a verbal pain scale. Written, informed consent in accordance with the institutional review board was obtained prior to the start of the testing session.

### 2.2. Application of Spinal Manipulation

A total of 2 lumbar diversified side-lying high-velocity low-amplitude (HVLA) manipulations and 2 side-lying grade-IV mobilizations were performed on each participant by one of two chiropractors, each with over 10 years of experience. One manipulation and one mobilization were performed at the L3 spinal level and one at the SI level, using a hypothenar hand contact. HVLA manipulations consisted of a single quick force, while the mobilizations consisted of 5 slower, less forceful rocking motions delivered at a frequency of 1 Hz. The order of treatments was randomized, the time between the treatments was between 1 and 3 min, and only the data from the HVLA manipulations were used in this analysis.

### 2.3. EMG and Force Instrumentation during Manipulation

**Figure 1:**
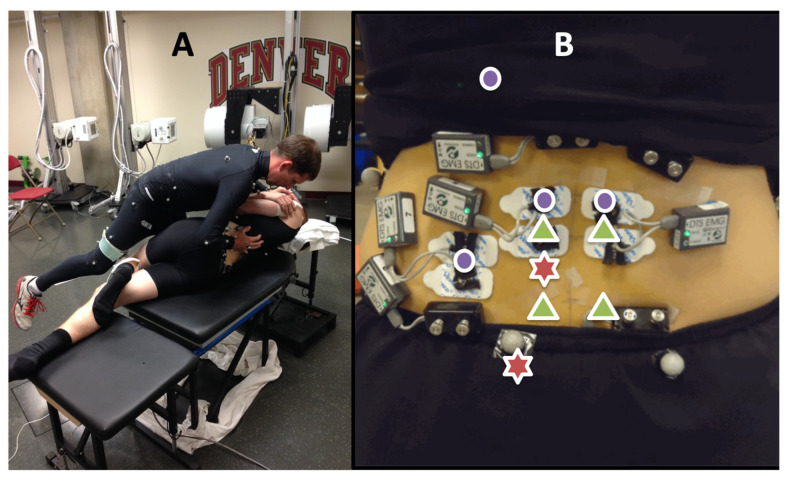
(**A**) Side-posture SM set-up. (**B**) Instrumentation included indwelling EMG (green triangles) and surface EMG (purple dots). Red stars indicate manipulation sites at the L3 and SI spinal levels on the left.

Eight EMG electrode pairs were used to record signals from the low back. Four surface electrodes were used, recording muscle activity from the erector spinae (bilaterally at the L2 spinal level), the lower trapezius (left), and the quadratus lumborum (left). A total of 4 indwelling electrodes (50 mm, 25 ga needle for insertion, with a pair of 0.051 mm, insulated, hooked wires, and a 200 mm tail with 5 mm bare-wire terminations) recorded muscle activity from the multifidi (L2 and L5 bilaterally) and were inserted 2.5 cm lateral and 1 cm superior to the tip of the spinous process at a 45 degree angle towards the spine [28]. A Noraxon TeleMyo DTS (Noraxon USA, Scottsdale, AZ, USA) system was used to record both surface and indwelling EMG signals. The force from the practitioner’s contact hand was estimated using an optimized algorithm that combined measurements from force transducers attached to the practitioner with measurements from a force plate (Bertec Corporation, Columbus, OH, USA) embedded in the treatment table, and allowed the maintenance of unimpeded contact between the practitioner and participant [29] (Figure 1).

### 2.4. EMG Signal Processing

Movement artifact and high frequency noise were removed from the raw EMG signals with a bandpass filter (4th-order Butterworth, 15–350 Hz). The signals were transformed using the Teager Kaiser Energy Operator (TKEO), which improved muscle activity onset detection [30,31].

### 2.5. Muscle Response and Muscle Activity Onset Delay

The presence of a muscle response was determined with a double-threshold method that contained amplitude and duration components optimized specifically for the HVLA manipulations [32]. Muscle response was calculated by recording the number of locations with a response and dividing this by the total number of muscle locations evaluated, expressed as a percentage (Equation (1)).

(1) $$Muscle\;Response\;=\;\frac{\#\;of\;Positive\;Responses}{\#\;of\;Muscle\;Locations}\times 100$$

Muscle activity onset delay, a timing variable, was calculated as the time delay between the first positive slope of the manipulation force and the first energy transformed EMG activity that resulted in a muscle response.

### 2.6. Analysis of Treatment and Response Factors

The analysis of dependent variables was separated into two categories: (1) *treatment factors*, which focused on how the SM was applied, and (2) *response factors,* which focused on the type and location of the muscles that responded to the SM. 

The effect of the *treatment factors*, including manipulation sequence (first or second) and manipulation site (L3 or SI), on the number of muscle responses and the muscle activity onset delays were compared using mixed-model linear regressions with a random effect for the subject. The equivalence values between the first and second manipulations were determined for both muscle response and muscle activity onset delay by using a varying practical difference threshold until the equivalences were achieved.

The effect of the *response factors*, including muscle location (indwelling L2, L5, surface L2, QL, and trapezius), muscle layer (multifidus or superficial), and muscle side (left or right), on the number of muscle responses and the muscle activity onset delays were compared using mixed-model linear regression with a random effect for the subject. Only muscles with a contralateral analog were used for right and left comparisons (L2 multifidus, L5 multifidus, and L2 erector spinae). Effect sizes—which expressed the difference between two population means as a percentage of one standard deviation, and were a measure of the magnitude of the effect [33]—were calculated for all comparisons using Cohen’s *d*. We chose the following scale based on practical definitions: a small effect was defined as 0.2 < *d* ≤ 0.5, a moderate effect as 0.51 ≤ *d* ≤ 0.79, and a large effect as *d* ≥ 0.8 [34].

## 3. Results

### 3.1. Treatment Factors

#### 3.1.1. Manipulation Sequence

For all muscle locations, a response occurred in 67.5 ± 26.7% of the first manipulations, and 55.6 ± 29.9% of the second manipulations. The effect size of manipulation order was small (*d* = 0.42), and the difference was not statistically significant (*β* = −11.8%, SE = 7.1%, *p* = 0.11). Where *β* was the model-predicted regression offset of the second manipulation, a negative *β* value indicated a lower value for the second manipulation. There were no effect sizes and no statistically significant differences in the muscle activity onset delays between the first and second manipulations (*β* = −10.1 ms, SE = 11.7 ms, *p* = 0.35). Equivalence testing revealed the first and second manipulations were equivalent at the 28% practical difference threshold for the muscle responses, and equivalent at the 30 ms practical difference threshold for the muscle activity onset delays.

#### 3.1.2. Manipulation Site

For all muscle locations, a response occurred in 65.6 ± 24.3% of the L3 manipulations and 57.5 ± 32.5% of the SI manipulations. The effect size of the manipulation site was small (*d* = 0.29) and the difference was not statistically significant (*β* = −4.1%, SE = 3.7%, *p* = 0.28), where *β* was the model-predicted regression offset of the SI manipulation. There were no effect size and no statistically significant difference in the muscle activity onset delays in all muscles between the L3 and SI manipulation sites (*β* = −5.5 ms, SE = 5.8 ms, *p* = 0.35).

### 3.2. Response Factors

#### 3.2.1. Muscle Layer

**Figure 2:**
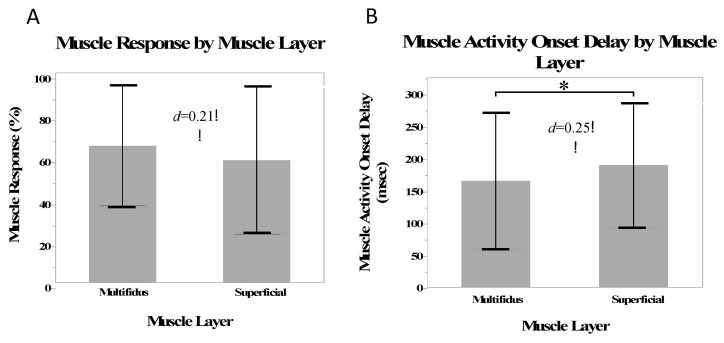
Mean muscle responses (**A**) and mean muscle activity onset delay (**B**) for the multifidus and superficial muscles. Stars indicate a statistically significant difference.

There was a small effect size (*d* = 0.20) and no statistical difference between the multifidus and superficial muscles in the number of muscle responses (*β* = 3.4%, SE = 2.83%, *p* = 0.23). There was a small effect size (*d* = 0.25) and significantly shorter muscle activity onset delays in the multifidi than in the superficial muscles (*β* = −13.0 ms, SE = 5.6 ms, *p* = 0.02) (Figure 2).

#### 3.2.2. Muscle Location

**Figure 3:**
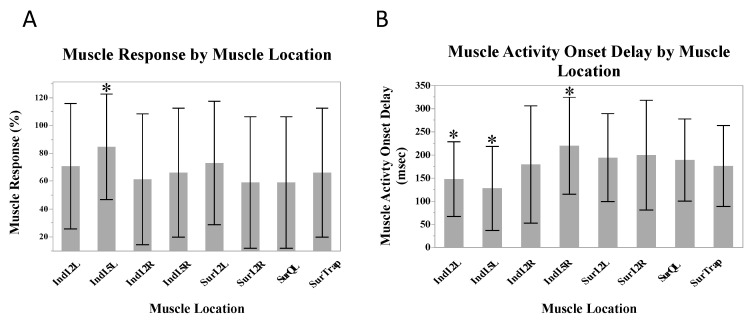
Mean muscle responses (**A**) and mean muscle activity onset delays (**B**) across all muscle locations. Stars indicate statistically significant differences from the mean.

A greater number of responses occurred in the L5 indwelling location on the left, compared to the other muscle locations (*β* = 18.4%, SE = 6.5%, *p* = 0.047). Shorter muscle activity onset delays occurred in the L2 (*β* = −27.7 ms, SE = 13.9 ms, *p* = 0.047) and L5 (*β* = 55.9 ms, SE = 12.8 ms, *p* = 0.001) indwelling electrodes on the left, and longer delays occurred in the L5 indwelling electrode on the right (*β* = 38.8 ms, SE = 14.4 ms, *p* = 0.01) (Figure 3).

**Figure 4:**
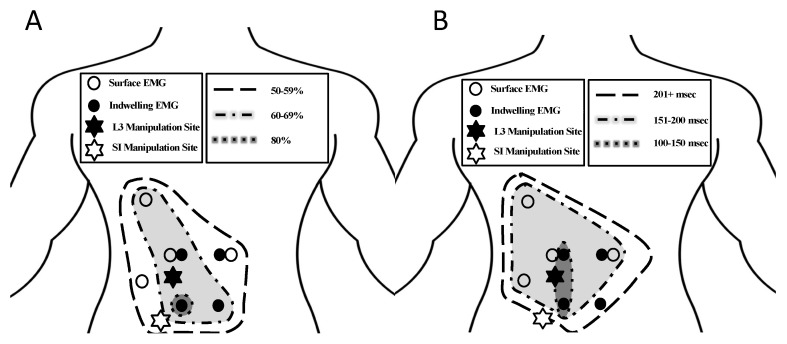
Anatomical distribution of muscle responses (**A**) and muscle activity onset delays (**B**) for all 8 muscle locations in relation to the manipulation locations (indicated by stars).

The muscle response and muscle activity onset delays revealed a pattern of decreasing muscle responses and increasing muscle activity onset delays as the distance from the manipulation location increased (Figure 4).

#### 3.2.3. Muscle Side

**Figure 5:**
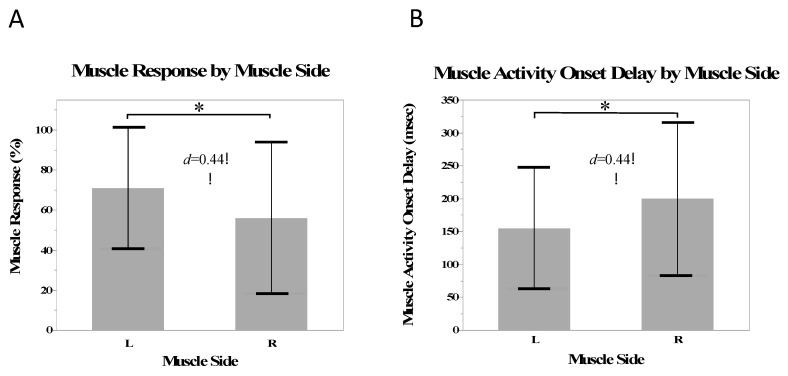
Mean muscle responses (**A**) and mean muscle activity onset delay (**B**) for the on the left and right side. Stars indicate a statistically significant difference.

There was a small effect size of side on the muscle response (*d* = 0.44) and a greater number of responses in the muscles on the left side than the right side (*β* = 7.5%, SE = 3.0%, *p* = 0.02). There was a small effect size (*d* = 0.44) and significantly shorter muscle activity onset delays in the muscles on the left side compared with the right (*β* = −23.0 ms, SE = 6.7 ms, *p* < 0.001) (Figure 5).

## 4. Discussion

This study quantified the effects of *treatment* and *response factors* on the neuromuscular responses to SM as measured by the rate of muscle responses and muscle activity onset delays. Factors that may have had an effect on the responses using a standardized method were identified and the neuromuscular responses in a healthy population *during* a spinal manipulation were quantified for eight important back muscles. These results suggest that future studies and clinical treatment focusing on timing outcomes in healthy participants can be designed without regard for manipulation sequences (separated by at least 3 min) and locations (at least within the lumbar region), whereas studies focusing on the number of muscle responses may want to consider these variables due to the small effects noted.

The lack of a statistically significant difference between the first and second manipulations could be viewed as an indicator of confidence in the repeatability of the measures; noteworthy, however, the muscle response was equivalent at a 28% difference threshold, which was high enough to be impractical for considering these responses equivalent, and the muscle activity onset delays could be considered equivalent at the practical level of 30 ms. This result is consistent with the finding of a small effect of the sequence for the muscle responses and no effect on the muscle activity onset delays. In this study, the first and second manipulations were separated by 1–3 min and were in close anatomic proximity (L3 and SI). Although we were unable to find investigations on reliability for EMG measures in the spinal manipulation literature, the within-session reliability for EMG measures in the low back during active tasks was moderate to high [35]. The activation of a motor unit depends on multiple factors, including the type of muscle, training level of the participant, and fatigue state of the muscle [36], making comparisons across days and muscle groups difficult [37]. This study was not statistically powered to evaluate the differences between first and second manipulations; however, a consideration for an effect of sequence in future studies should be considered, especially those concerned with the number of muscle responses.

The lack of statistical differences in the neuromuscular responses during SM performed at different manipulation sites demonstrated that the specificity of the contact site may not have play a major role in the biomechanical outcomes. The close proximity of the two manipulation sites may explain the lack of differences in this study and is consistent with other location variables analyzed during SM. Previous work has found no difference in the responses, such as joint cavitation, regardless of whether the manipulation was directed at the L5 or SI joints [38]; that responses could occur above and below the targeted joint [39]; and that hand configuration could lead to forces being applied to levels other than the targeted vertebrae [40]. Given the broad spinal, pelvic, and thigh motions produced during a side-lying manipulation and the size of the hand contact relative to the anatomical structures being manipulated, it was not surprising that the neuromuscular responses were not affected by a small difference in a single contact point.

This study reported the previously undocumented response in the multifidus *during* a side-lying SM. Shorter onset delays in the multifidus indicated a faster response than the erector spinae and that this muscle could not be considered similar to others for comparisons across the research studies. Previous work has focused on the muscle response of the erector spinae as it is relatively easy to measure, leaving gaps in the knowledge regarding deeper muscles, such as the multifidus, which is important for spinal stability. Because of its deep location and proximity to the vertebral bodies that are a target of spinal manipulation, the multifidus is often the first muscle to respond to SM. The increased responses and shorter onset delays seen in the multifidus may be a characteristic of this healthy population, and future comparisons to low-back-pain participants are warranted to determine if SM can play a role in multifidus activation.

The trend of decreasing muscle response rates and longer muscle activity onset delays as measurements moved farther away from the site of manipulation (Figure 4) indicates a relation of muscle response to manipulation with characteristic spatial and timing patterns. Muscle responses at distant locations were present, which was consistent with the previous findings in lumbar and SI manipulations using surface EMG [13]; however, this work added the relation of muscle response and muscle activity onset delays to manipulation location, which has not been seen in the previous work. This spatial pattern of the left-sided multifidus having the greatest muscle response and the shortest onset delays in the presence of a left-sided manipulation may be a result of the novel addition of the indwelling multifidus recordings in this study. The understanding of the neuromuscular response to SM is improved by the identification of a characteristic timing pattern in response to SM allowing for future comparisons for participants in pain.

The greater muscle response and shorter delays on the left side compared to the right were consistent with the stretch of the tissues on the upper side of the patient during a side-lying manipulation. The left-side (up-side) muscles responded at a greater rate and had a faster response, which was consistent with the trend of a greater effect size of SM closer to the treatment site. Interestingly, the multifidi as a whole group did not always have a greater number of responses or the shortest onset delays; however, the left side multifidi did, indicating that the side of the response may have had more of an influence than the layer of the muscle responding (Figure 4). Because of the rotational nature of the side-lying manipulation, the up-side musculature was likely stretched more than the down side. The up side is also thought to receive the most force and has been shown to produce the most gapping of the facet joints [41]. Because tissues, such as the erector spinae, the multifidus, and the facet joint capsules are stretched during spinal manipulation [42], the muscle spindles and golgi tendon organs are stimulated [43] causing changes in the neuromuscular response. 

In the previous work using lumbar side-lying manipulation [13], the down-side muscles were not recorded; however, a greater number of responses were observed in the right-sided muscles when treatment was applied to the right side than in the left-sided muscles when treatment was applied to the left side. During a prone manipulation of an exposed vertebrae with a mechanical instrument, the right-sided nerve roots produced a greater number of responses [14]. The experimental set-up used in this study allowed measurements from both sides simultaneously for a direct comparison. Treatment was not applied to the right side, perhaps explaining why we did not have a 100% response rate for any measurement where the previous work did (i.e., we measured the least responsive side). It is unclear if the greater responses on the right side in the previous literature are due to a side dominance of the participant, or a side dominance in the delivery of the manipulation. 

A limitation of this study is that the manipulation was not directed at a specific clinical lesion or hypertonic muscle, but rather predetermined by the study design—assigned randomly to the L3 spinal level or the SI. This assignment allowed for an unbiased comparison of the muscle responses and timing between standardized treatment sites. Some evidence exists that directing treatment toward a clinical lesion produces less variable EMG amplitude changes [11] than when one where the treatment is directed toward a specific vertebral level [16]. 

## 5. Conclusions

*Treatment factors* (manipulation sequence and site) had little effect on the neuromuscular response to manipulation, indicating that researchers and clinicians may choose to design protocols without regard for these factors as the specificity of the contact site may not play a major role in the biomechanical outcomes, at least within the lumbar spine area. The neuromuscular response to manipulation did not change significantly as multiple manipulations were performed at similar sites (SI joint and L3). *Response factors*, including the location, layer, and side of the neuromuscular response revealed trends of decreasing muscle response rates and increasing muscle activity onset delays as the distance from the manipulation location increased. Clinical treatment protocols and future research studies may be designed with these results in mind, when choosing the application site for treatment and the locations for EMG measurements. We anticipate the future work to compare subjects in pain, with correlations to clinical outcome measures to aide in the mechanistic understanding of the neuromuscular response to SM.

## Figures and Tables

**Table 1:** Mean ± SD participant anthropometric information.

	Male (n = 10)	Female (n = 10)
Age (years)	33.4 ± 13.9	31.8 ± 7.9
Height (cm)	179.3 ± 7.3	165.0 ± 2.9
Weight (kg)	79.3 ± 9.0	60.2 ± 5.0
Dominant hand (right/left)	(9/1)	(8/2)

## Data Availability

The datasets used and/or analyzed during the current study resulted from a dissertation project at the University of Denver [44] and are available.
